# Small-study effects and time trends in diagnostic test accuracy meta-analyses: a meta-epidemiological study

**DOI:** 10.1186/s13643-015-0049-8

**Published:** 2015-05-09

**Authors:** Wynanda Annefloor van Enst, Christiana A Naaktgeboren, Eleanor A Ochodo, Joris AH de Groot, Mariska M Leeflang, Johannes B Reitsma, Rob JPM Scholten, Karel GM Moons, Aeilko H Zwinderman, Patrick MM Bossuyt, Lotty Hooft

**Affiliations:** Department of Clinical Epidemiology, Biostatistics and Bioinformatics, Academic Medical Center, University of Amsterdam, Meibergdreef 9, 1105 AZ Amsterdam, Netherlands; Dutch Cochrane Centre, Julius Center for Health Sciences and Primary Care, University Medical Center Utrecht, University of Utrecht, Universiteitsweg 100, 3584 CG Utrecht, Netherlands; Julius Center for Health Sciences and Primary Care, University Medical Center Utrecht, University of Utrecht, Universiteitsweg 100, 3584 CG Utrecht, Netherlands; Centre for Evidence-based Health Care, Faculty of Medicine and Health Sciences, Stellenbosch University, Francie van Zijl Drive, Tygerberg, PO Box 241, Cape Town, 8000 South Africa

**Keywords:** Diagnostic test accuracy, Sensitivity, Specificity, Meta-analyses, Publication bias, Small-study effects, Time trends, Systematic reviews

## Abstract

**Background:**

Small-study effects and time trends have been identified in meta-analyses of randomized trials. We evaluated whether these effects are also present in meta-analyses of diagnostic test accuracy studies.

**Methods:**

A systematic search identified test accuracy meta-analyses published between May and September 2012. In each meta-analysis, the strength of the associations between estimated accuracy of the test (diagnostic odds ratio (DOR), sensitivity, and specificity) and sample size and between accuracy estimates and time since first publication were evaluated using meta-regression models. The regression coefficients over all meta-analyses were summarized using random effects meta-analysis.

**Results:**

Forty-six meta-analyses and their corresponding primary studies (*N* = 859) were included. There was a non-significant relative change in the DOR of 1.01 per 100 additional participants (95% CI 1.00 to 1.03; *P* = 0.07). In the subgroup of imaging studies, there was a relative increase in sensitivity of 1.13 per 100 additional diseased subjects (95% CI 1.05 to 1.22; *P* = 0.002). The relative change in DOR with time since first publication was 0.94 per 5 years (95% CI 0.80 to 1.10; *P* = 0.42). Sensitivity was lower in studies published later (relative change 0.89, 95% CI 0.80 to 0.99; *P* = 0.04).

**Conclusions:**

Small-study effects and time trends do not seem to be as pronounced in meta-analyses of test accuracy studies as they are in meta-analyses of randomized trials. Small-study effects seem to be reversed in imaging, where larger studies tend to report higher sensitivity.

**Electronic supplementary material:**

The online version of this article (doi:10.1186/s13643-015-0049-8) contains supplementary material, which is available to authorized users.

## Background

The validity and credibility of the results of a systematic review of diagnostic test accuracy studies depend not only on the methodological quality of the included studies but also on the absence of selective reporting [1-3]. Knowledge about the principles of selective reporting can help with the interpretation of the results of a meta-analysis.

A sample-size effect in randomized trials has been described before. Published trials with smaller sample sizes tend to have larger and more favourable effects compared to studies with larger sample sizes [4,5]. This phenomenon may occur for several reasons. It has been suggested that smaller studies are more likely to be published when they show significant positive results. Larger studies may more likely be submitted, accepted and published regardless of their estimated effect. This mechanism, which is called small-study effect, can hamper the validity of a systematic review by overestimating the ‘true’ effect [3,6-8].

In addition to a small-study effect, meta-analyses of randomized trials may also be influenced by the problems arising from a time-lag effect. This effect can result from variability in the time it takes to complete and publish a study report, which may depend on the direction and strength of the trial results [9]. Empirical studies have indicated that negative or null results take approximately two or three more years to be published compared to positive results [3,10]. This time-lag effect could influence the meta-analysis and has implications for the timing of a review, the inclusion of on-going studies, and for updating the review.

Studying time-lag effects in diagnostic studies is not currently possible due to the lack of registration of such studies. However, overarching time trends, the tendency for study findings to change over time within a review, can be used as a proxy for time-lag effects. Although time-lag effects are only one reason for a change in results over time within a review, discovery of time trends would increase concern about the possible presence of time-lag effects in diagnostic accuracy studies.

Whereas these effects are well known and described for randomized trials, it is unclear whether they also translate to diagnostic accuracy studies [11-13]. Publication of diagnostic accuracy studies may be influenced by a different set of factors than randomized trials. In general, test accuracy studies tend to rely less on statistical significance testing than randomized trials. Many studies do not report confidence intervals around estimates [14], and sample size calculations based on a minimally relevant performance levels are typically absent [15]. However, there is some evidence of a failure to publish completed research projects. Korevaar *et al.* compared registry information for test accuracy studies to published reports and concluded that failure to publish and selective reporting are also present in test accuracy studies [16]. However, the mechanisms driving these in test accuracy studies are not understood.

In this study we aimed to assess whether meta-analyses of diagnostic test accuracy suffer from small-study effects or time trends, using a set of recently published systematic reviews of such studies.

## Methods

### Selection of reviews and meta-analyses

This study was part of a meta-epidemiological project on systematic reviews of diagnostic accuracy studies. On 12 September 2012, MEDLINE and EMBASE were searched for systematic reviews on test accuracy studies published between 1 May 2012 and 11 September 2012. For our analysis, we limited inclusion to reviews with a meta-analysis for which we were able to obtain all two-by-two classification tables of the studies included in the meta-analysis. A meta-analysis was defined as an analysis producing a summary estimate for at least one accuracy statistic or, alternatively, producing a summary ROC curve (sROC). Reviews of tests in animals, of prognostic tests, and of individual patient data were excluded, as there may be other effects related to publication in these types of studies. Only English language reviews were included. The full text of the search strategy is available in Additional file 1.

### Data extraction

Data were extracted using an online structured data-extraction form. An independent double data-extraction pilot was performed for a subset of the reviews (30%) until all authors agreed on the items of the data-extraction form. After that, data were extracted by one reviewer (CN, EO or WvE) and checked by a second reviewer (CN, EO or WvE) for discrepancies. Disagreements were resolved during a consensus meeting.

For each eligible review, we classified the type of test under evaluation and the total number of studies included in the meta-analyses. Data were then collected on the primary studies within one meta-analysis for each included review. Only one meta-analysis per review was included, so as not to give reviews with multiple meta-analysis extra weight and to avoid having to deal with correlated results. We selected the meta-analysis with the largest number of included primary studies, as the power to detect an association (if present) will be generally larger in meta-analyses with more primary studies. We assumed that there is no association between the number of studies in a meta-analysis and the associations of interest. For each primary study in a meta-analysis, we extracted the year of publication and data to populate the individual two-by-two accuracy table: the number of true positives, false negatives, false positives, and true negatives.

Whenever information on the primary studies was not available to us directly from the published review, we contacted the review authors. When we were unable to reach the author after sending two reminders or when authors could not provide the data, data were extracted from the original primary study reports. Failure to obtain this data from all studies in the meta-analysis was not a reason to exclude a meta-analysis. A second author checked the results of the data extraction.

### Data analysis

The aim of the analysis was to investigate the strength of the association between estimates of accuracy and sample size and between accuracy and time since first publication within a meta-analysis. These analyses were done in two steps. We first examined these associations within each included meta-analyses separately and then calculated a pooled estimate across all meta-analyses. This two-step approach was chosen to accommodate for differences in accuracy between meta-analyses related to differences in tests or fields.

These associations were examined for three commonly used measures of accuracy: sensitivity, specificity and the diagnostic odds ratio [12,17,18]. To examine the association between sensitivity and sample size (that is the number of diseased subjects in a study), we performed a random effects meta-regression using logit sensitivity as the outcome and including the number of diseased subjects as a covariate in the model. To account for differences in the precision of sensitivity estimates between studies, we used the exact binomial distribution to model the within-study sampling error. This means that larger studies (that is studies with more diseased subjects) get more weight in the analysis. We used the exact binomial distribution (that is a non-linear mixed model) to avoid the need to add 0.5 to the data, which would have produced a downward bias in estimates which is more pronounced in smaller studies [19,20]. The slopes and corresponding standard errors for sample size were estimated for each meta-analysis.

To prevent estimation problems when fitting this model, we excluded meta-analyses with four or fewer primary studies. In addition, primary studies without any diseased subject were also excluded from the analysis. To estimate the association across all meta-analyses, we then used the generic inverse variance method to get a pooled random effect estimate of this slope and its 95% confidence interval [21]. The analysis for the association of specificity and sample size was done in the same way, only now using the number of non-diseased subjects as the measure of sample size.

To examine the association between the magnitude of the diagnostic odds ratio and total sample size, we first estimated the natural logarithm of the odds ratio in each individual study using Firth’s correction method for small samples sizes [22]. This correction method is preferred as it avoids the need of adding 0.5 in case the odds ratio estimate cannot be calculated. This method also reduces the bias that exists when estimating the odds ratio in small samples [23]. We then fitted a meta-regression model for each meta-analysis separately using the log of the diagnostic odds ratio as the outcome and total sample size as the covariate. The inverse of the variances of the log odds ratios were used as weights (that is the generic inverse variance method was used) [21]. Similar to the analysis of sensitivity and specificity, the slopes were estimated within each meta-analysis and then combined across the meta-analyses.

Similar analyses were done to look at associations between accuracy and time since first publication in a meta-analysis. Here the time interval between the date of publication of each study and the date of the oldest publication within a meta-analysis was used as the covariate in the regression model.

Both sample size and time since first publication were entered as continuous covariates to the model, assuming a linear association. To examine whether there were non-linear associations, we also classified studies in each meta-analysis into three groups using tertiles of sample size and tertiles of time since the first publication in years, respectively, and used these tertiles as categorical variables in the meta-regression.

The non-linear mixed effects models for (logit) sensitivity and specificity were performed in SAS using the NLMIXED procedure [24]. All other analyses were conducted in the statistical package R [25].

### Subgroup analysis

In addition, separate analyses were carried out for imaging tests and for laboratory tests. Our rationale for this subgroup analysis was based on the observation that imaging studies generally have an implicit threshold. The reported accuracy in studies with an implicit threshold can be affected by the number of diseased patients and is more likely to change over time [26-28]. In addition, with imaging, gradual improvements in techniques may also induce time trends. We therefore hypothesized that a small study effect or time trends might act differently in imaging studies than in laboratory studies.

## Results

### Search results

The search identified 1,273 references. After screening the titles and abstracts, 89 references were found potentially eligible and were read as full-text articles. Attempts were made to obtain the two-by-two tables of 53 eligible reviews. In three reviews, attempts were unsuccessful in extracting all of the two-by-two tables, resulting in 50 reviews (Additional file 2). In four reviews, the number of primary studies was four or lower and therefore excluded from the meta-epidemiological analyses. The remaining 46 meta-analyses contained a total of 859 primary studies (Additional file 3 contains a list of the reviews).

### Characteristics of the included reviews and meta-analyses

Fourteen reviews investigated a laboratory test, 27 an imaging test and 5 addressed clinical examinations. The selected meta-analyses had a median of 14 primary studies (interquartile range (IQR) 10–20). The median prevalence of the target condition in the studies was 47% (IQR: 24%-69%). More characteristics of the primary studies are presented in Table 1.

**Table 1:** **Characteristics of primary studies (** ***N*** **= 859) across and within the included meta-analyses (** ***N*** **= 46)**

**Number of sample size**	**Median**	**Interquartile range**	**Min-max**
Across all meta-analyses			
Number of diseased	33	17-63	0-1,358
Number of non-diseased	36	18-100	0-49,973
Sample size	87	45-185	3-50,008
Time since first publication (years)	6	3-10	0-42
	Median of the median	Interquartile range of medians	Min-max of the median
Within meta-analyses			
Number of diseased	31	18-41	5-154
Number of non-diseased	38	18-87	0-11,276
Sample size	94	46-332	20-11,281
Time since first publication (years)	5	4-11	0-26

### Sample size

The median sample size of the included studies (*N* = 859) was 87 participants (IQR 45–185), ranging from extremely small (*N* = 3) to very large (*N* = 50,008). In total, there were 51,875 diseased participants and 525,838 non-diseased. This skewed distribution was mainly caused by a small set of studies on screening tests with very large samples but very few diseased compared to non-diseased.

The relative increase in DOR per 100 participants was 1.01 (95% CI 1.00 to 1.03; *P* = 0.07). This increase was mainly due to a relative increase in sensitivity of 1.11 per 100 additional diseased subjects (95% CI 0.98 to 1.26; *P* = 0.09). The analyses by tertiles confirmed that larger studies tend to produce higher estimates of accuracy, in particular for sensitivity (see Table 2).

**Table 2:** **Small-study effects and time trends**

	**Accuracy measure** ^**a**^	**Relative increase** ^**b**^ **(95% CI)** ***P*** **value**	**T2 vs. T1** ^**c**^ **(95% CI)** ***P*** **value**	**T3 vs. T1** ^**c**^ **(95% CI)** ***P*** **value**
Number of diseased	Sensitivity	1.11 (0.98 to 1.26) *P* = 0.09	1.08 (0.87 to 1.34) *P* = 0.50	1.22 (0.99 to 1.51) *P* = 0.06
Number of non-diseased	Specificity	1.00 (0.99 to 1.02) *P* = 0.49	1.05 (0.83 to 1.33) *P* = 0.66	0.97 (0.73 to 1.28) *P* = 0.82
Sample size	DOR	1.01 (1.00 to 1.03) *P* = 0.07	1.15 (0.94 to 1.40) *P* = 0.16	1.26 (0.96 to 1.64) *P* = 0.09
Time since first publication	Sensitivity	0.89 (0.80 to 0.99) *P* = 0.04	0.61 (0.11 to 3.39) *P* = 0.57	0.59 (0.13 to 2.67) *P* = 0.49
Time since first publication	Specificity	1.04 (0.90 to 1.19) *P* = 0.60	1.07 (0.85 to 1.35) *P* = 0.55	1.00 (0.76 to 1.32) *P* = 0.99
Time since first publication	DOR	0.94 (0.80 to 1.10) *P* = 0.42	0.94 (0.71 to 1.25) *P* = 0.68	0.93 (0.71 to 1.21) *P* = 0.57

^a^The analyses were performed on the natural logarithm of the DOR and on the logit scale for sensitivity and specificity; ^b^relative increase for sensitivity, specificity, and DOR is reported per increase in 100 diseased, non-diseased, or total participants, respectively. For time since first publication, the relative increase is reported per 5 year increase; ^c^T1 is the lowest tertile of sample size or time since first publication, T3 the highest. DOR, diagnostic odds ratio.

### Time of publication

The primary studies included in the meta-analyses were published between 1969 and 2012. Within meta-analyses, the median time interval between the first and the last included publication was 6 years (IQR: 3–10). For the DOR, there was a non-significant, negative association with time since first publication in a review, with a relative decrease in DOR of 0.94 per 5 years (95% CI 0.80 to 1.10; *P* = 0.42). Again this was mainly caused by the trend observed for sensitivity, with a relative decrease per 5 year of 0.89 (95% CI 0.80 to 0.99; *P* = 0.04. No association between time since first publication and specificity was observed. For detailed results see Table 2.

### Subgroup analysis

The subgroup analyses by type of test (that is imaging technique or laboratory test) revealed that the observed associations in the overall group were mainly caused by studies examining an imaging technique. In particular, a highly significant and positive association was found between sample size and sensitivity in imaging studies. In other words, larger imaging studies tend to report higher values of sensitivity than smaller studies examining the same technique. For detailed results see Table 3.

**Table 3:** **Small-study effects and time trends assessed in subgroups of imaging and laboratory tests**

	**Accuracy measure** ^**a**^	**Imaging test (** ***N*** **= 27 meta-analyses)**	**Laboratory test (** ***N*** **= 14 meta-analyses)**
		**Relative increase** ^**b**^ **(95% CI)** ***P*** **value**	**Relative increase** ^**b**^ **(95% CI)** ***P*** **value**
Diseased	Sensitivity	1.13 (1.05 to 1.22) *P* = 0.002	1.01 (0.80 to 1.28) *P* = 0.92
Non-diseased	Specificity	1.03 (0.86 to 1.24) *P* = 0.73	1.01 (1.00-1.02) *P* = 0.14
Sample size	DOR	1.05 (0.97 to 1.14) *P* = 0.23	0.94 (0.63 to 1.40) *P* = 0.76
Time since first publication	Sensitivity	0.88 (0.77 to 1.00) *P* = 0.05	0.94 (0.75 to 1.17) *P* = 0.57
Time since first publication	Specificity	1.02 (0.87 to 1.19) *P* = 0.84	1.13 (0.80 to 1.58) *P* = 0.50
Time since first publication	DOR*	0.89 (0.78 to 1.02) *P* = 0.09	0.87 (0.42 to 1.81) *P* = 0.71

^a^The analyses were performed on the natural logarithm of the DOR and on the logit scale for sensitivity and specificity; ^b^relative increase for sensitivity, specificity, and DOR is reported per increase in 100 diseased, non-diseased, or total participants, respectively. For time since first publication, the relative increase is reported per 5 year increase. *DOR, diagnostic odds ratio.

## Discussion

We assessed the existence of small study effects and time trends in test accuracy meta-analyses using a meta-epidemiological analysis of a series of published systematic reviews. Opposite to what was expected, we observed no significant effects, and accuracy estimates of diagnostic studies tended to be lower in studies with a small sample size compared to studies with a larger sample size. Time trends were in the opposite direction; studies published after the first publications tended to report lower sensitivity. These trends were mainly caused by the subgroup of studies examining an imaging technique.

Our findings are in contrast with earlier findings for meta-analyses of randomized trials, where higher treatment effect sizes were reported to be strongly associated with small sample sizes [7,8,29]. Nüesch *et al.* studied 13 meta-analyses with continuous outcomes and found on average a 0.21 (95% CI 0.08 to 0.34) higher effect size in small trials than in large trials [7]. Dechartres *et al.* included 93 meta-analyses with binary outcomes. They concluded that the quartile of smallest trials had 32% (95% CI 18% to 43%) larger treatment effects than the quartile with the largest trials [8]. The differences in effect size between small and large trials can be the result of the publication process, which selects positive and significant results over negative or null results [30]. According to the review of Hopewell *et al.*, the odds of finding positive, significant results in a publication are four times higher than finding negative or null results [31].

We consider it unlikely that the sample-size effect we have found for test accuracy meta-analyses results from an actual preference to publish small studies with low accuracy estimates, rather than small studies with higher accuracy estimates. Our findings on sample size effect are similar to those of Haines and colleagues. They found a similar relation between sample size and Youden’s Index, a test statistic that captures test performance [32]. In their evaluation, studies with larger samples had a higher Youden’s Index. Haines *et al.* claimed that this relationship was attributable to prematurely stopping studies with poorer outcomes at smaller sample sizes while still publishing the results. It will be challenging to assess if this hypothesis is valid because power calculations are rarely reported in diagnostic accuracy studies [33]. Another possibility is that the small-sample effect is a result of variability in methodological quality. In diagnostic research, some large studies could be based on routine care data. Such data may suffer from verification problems, resulting in higher accuracy [34]. In any case, the mechanisms behind the small-study effects observed deserve further exploration.

Another issue that deserves attention when meta-analysing estimates of accuracy is when zero counts occur in the two-by-two table; in such cases, sensitivity or specificity estimates are 0% or 100%, or the odds ratio estimate is infinity. A standard solution is to add 0.5 to each cell of the two-by-two table [35]. Although this correction prevents estimation problems, it does produce a downward bias in the estimates, which is more pronounced in small samples. To avoid imposing a positive association between the level of accuracy and sample size, it is critical to avoid the 0.5 correction. For this reason, we use non-linear mixed models to meta-analyse the logit transformed sensitivities or specificities within each review, with the exact binomial distribution to model the within-study sampling error [19,20]. For our analyses with the odds ratio as the outcome, we estimate the log of the odds ratio in each individual study by using Firth’s correction method [22] to avoid the need of adding 0.5 [23].

Our primary analysis was based on the assumption of a linear relationship between test accuracy and sample size or time since first publication. We assumed that if an association between sample size or time since first publication is present, a more or less linear association is most likely. As the actual relation might deviate, we also performed an additional analysis by tertiles of sample size or time since first publication as a way to examine potential non-linear associations. These analyses revealed no indications of strong non-linear associations.

The time trend observed in our DTA meta-analyses was limited, compared to several time trends identified for randomized trials [3,9,35]. We observed a negative association between sensitivity and time after the first publication within a review. This direction is similar to the trend of randomized trials, with lower DORs in later studies. Similar to our results, the study of Sonnad *et al.* found that earlier published studies had higher accuracy, but the relation was not significant [36].

A possible explanation for such a trend is that the design of studies may change over time, from explorative case control type studies to prospective studies in consecutive patients [37]. In addition, the setting and targeted patients may change over time, with better understanding of the most useful application of a diagnostic test [38]. It would be worthwhile to study if specific study characteristics, such as study setting or patient spectrum, change over time in a large cohort of primary diagnostic accuracy studies. In future studies it would also be worthwhile to investigate the separate the effects of sample size and time trends by jointly modelling them.

The trends in our meta-epidemiological study were more pronounced for the sensitivity of imaging tests. The reasons for this remain speculative. Differences in the inclusion or exclusion criteria or setting of a diagnostic study are more likely to affect the case-mix of patients with the target condition and therefore have a greater effect on sensitivity. High-resolution imaging techniques are often used at the end of a diagnostic pathway and determine further clinical actions. Convincing evidence showing high accuracy in a large number of patients may therefore be pursued.

The meticulous follow-up of a cohort of diagnostic accuracy studies would be a way of documenting the actual mechanisms in designing, reporting and publishing such studies, and would allow us to analyse to what extent non-random publication bias exists [9,39]. Factors that influence the decision to submit or to accept a research article can also be studied from trial registers. In 2006, the International Committee of Journal Editors (ICMJE) established prospective registration of trials, defined as ‘any research project that prospectively assigns human subjects to intervention and comparison groups to study the cause-and-effect relationship between a medical intervention and a health outcome’ [40]. At present, this definition does not seem to capture all test accuracy studies, and recent analyses have shown that only a small subset of such studies is currently registered before enrolment of the first patient [41].

## Conclusions

Awaiting further evidence, our study results lead us to conclude that some of the typical mechanisms associated with publication bias, which are well documented in the literature for randomized clinical trials, are less prominent in test accuracy research. Small-study effects seem to be reversed in meta-analyses on imaging tests, where larger studies tend to report higher sensitivity. Confirmation of the findings of our study may provide reassurance to those relying on the published literature for evidence of the performance of medical tests.

## Additional files

Additional file 1:
**Full text of search strategy.** Full text search strategy used in this study to find reviews of diagnostic tests. Additional file 2:
**Inclusion flow chart.** Flow chart showing how reviews were included in this study. Additional file 3:
**List of included reviews.** References of reviews included in this study.

## References

[1] Dickersin K (1990). The existence of publication bias and risk factors for its occurrence. JAMA.

[2] Simes RJ (1986). Publication bias: the case for an international registry of clinical trials. J Clin Oncol.

[3] Song F, Parekh S, Hooper L, Loke YK, Ryder J, Sutton AJ, et al. Dissemination and publication of research findings: an updated review of related biases. Health Technol Assess. 2010;14(8):iii. ix-iii,193. 10.3310/hta14080 20181324

[4] Begg CB, Mazumdar M (1994). Operating characteristics of a rank correlation test for publication bias. Biometrics.

[5] Egger M, Davey Smith G, Schneider M, Minder C (1997). Bias in meta-analysis detected by a simple, graphical test. BMJ.

[6] Kjaergard LL, Villumsen J, Gluud C (2001). Reported methodologic quality and discrepancies between large and small randomized trials in meta-analyses. Ann Intern Med.

[7] Nuesch E, Trelle S, Reichenbach S, Rutjes AW, Tschannen B, Altman DG (2010). Small study effects in meta-analyses of osteoarthritis trials: meta-epidemiological study. BMJ..

[8] Dechartres A, Trinquart L, Boutron I, Ravaud P (2013). Influence of trial sample size on treatment effect estimates: meta-epidemiological study. BMJ..

[9] Ioannidis JP (1998). Effect of the statistical significance of results on the time to completion and publication of randomized efficacy trials. JAMA.

[10] Hopewell S, Clarke M, Stewart L, Tierney J (2007). Time to publication for results of clinical trials. Cochrane Database Syst Rev..

[11] Centre for R, Dissemination. Clinical tests. In: Systematic reviews: CRD’s guidance for undertaking reviews in health care. 2013.

[12] Macaskill P, Gatsonis C, Deeks JJ, Harbord RM, Takwoingi Y, Deeks JJ, Bossuyt PM, Gatsonis C (2010). Analysing and presenting results. Cochrane handbook for systematic reviews of diagnostic test accuracy: the Cochrane Collaboration.

[13] Deeks JJ (2001). Systematic reviews in health care: systematic reviews of evaluations of diagnostic and screening tests. BMJ.

[14] Korevaar DA, van Enst WA, Spijker R, Bossuyt PM, Hooft L. Reporting quality of diagnostic accuracy studies: a systematic review and meta-analysis of investigations on adherence to STARD. Med: Evid. Based; 2013. 10.1136/eb-2013-101637 24368333

[15] Bachmann LM, Puhan MA, ter Riet G, Bossuyt PM (2006). Sample sizes of studies on diagnostic accuracy: literature survey. BMJ.

[16] Korevaar DA, Ochodo EA, Bossuyt PM, Hooft L (2014). Publication and reporting of test accuracy studies registered in ClinicalTrials.gov. Clin Chem.

[17] Deeks JJ, Macaskill P, Irwig L (2005). The performance of tests of publication bias and other sample size effects in systematic reviews of diagnostic test accuracy was assessed. J Clin Epidemiol.

[18] Glas AS, Lijmer JG, Prins MH, Bonsel GJ, Bossuyt PM (2003). The diagnostic odds ratio: a single indicator of test performance. J Clin Epidemiol.

[19] Hamza TH, van Houwelingen HC, Stijnen T (2008). The binomial distribution of meta-analysis was preferred to model within-study variability. J Clin Epidemiol.

[20] Chu H, Cole SR (2006). Bivariate meta-analysis of sensitivity and specificity with sparse data: a generalized linear mixed model approach. J Clin Epidemiol.

[21] DerSimonian R, Laird N (1986). Meta-analysis in clinical trials. Control Clin Trials.

[22] Firth D (1993). Bias reduction of maximum likelihood estimates. Biometrika..

[23] Heinze G, Schemper M (2002). A solution to the problem of separation in logistic regression. Stat Med.

[24] The SAS system for Windows [program]. Release 9.2 version. Cary, NC: SAS Institute; 2011.

[25] Team RC (2013). R: a language and environment for statistical computing: R foundation for statistical computing.

[26] Willis BH (2012). Empirical evidence that disease prevalence may affect the performance of diagnostic tests with an implicit threshold: a cross-sectional study. BMJ Open.

[27] Dias-Silva D, Pimentel-Nunes P, Magalhaes J, Magalhaes R, Veloso N, Ferreira C (2014). The learning curve for narrow-band imaging in the diagnosis of precancerous gastric lesions by using web-based video. Gastrointest Endosc.

[28] Kheir F, Alokla K, Myers L, Palomino J. Endobronchial ultrasound-transbronchial needle aspiration of mediastinal and hilar lymphadenopathy learning curve. Am J Ther. 2014. 10.1097/MJT.0000000000000050 PMC4160423 24621644

[29] Shang A, Huwiler-Muntener K, Nartey L, Juni P, Dorig S, Sterne JA (2005). Are the clinical effects of homoeopathy placebo effects? Comparative study of placebo-controlled trials of homoeopathy and allopathy. Lancet.

[30] Dickersin K, Chalmers I (2011). Recognizing, investigating and dealing with incomplete and biased reporting of clinical research: from Francis Bacon to the WHO. J R Soc Med.

[31] Hopewell S, Loudon K, Clarke MJ, Oxman AD, Dickersin K (2009). Publication bias in clinical trials due to statistical significance or direction of trial results. Cochrane Database Syst Rev..

[32] Haines TP, Hill K, Walsh W, Osborne R (2007). Design-related bias in hospital fall risk screening tool predictive accuracy evaluations: systematic review and meta-analysis. J Gerontol A Biol Sci Med Sci.

[33] Carley S, Dosman S, Jones SR, Harrison M (2005). Simple nomograms to calculate sample size in diagnostic studies. Emerg Med J.

[34] Naaktgeboren CA, de Groot JA, van SM, Moons KG, Reitsma JB (2013). Evaluating diagnostic accuracy in the face of multiple reference standards. Ann Intern Med.

[35] Higgins JPT, Green S (editors). Cochrane handbook for systematic reviews of interventions version 5.1.0 [updated March 2011]. The Cochrane Collaboration. 2011. www.cochrane-handbook.org.

[36] Sonnad SS, Langlotz CP, Schwartz JS (2001). Accuracy of MR imaging for staging prostate cancer: a meta-analysis to examine the effect of technologic change. Acad Radiol.

[37] Lijmer JG, Leeflang M, Bossuyt PMM (2009). Proposals for a phased evaluation of medical tests.

[38] Irwig L, Bossuyt P, Glasziou P, Gatsonis C, Lijmer J (2002). Designing studies to ensure that estimates of test accuracy are transferable. BMJ.

[39] Stern JM, Simes RJ (1997). Publication bias: evidence of delayed publication in a cohort study of clinical research projects. BMJ.

[40] DeAngelis CD, Drazen JM, Frizelle FA, Haug C, Hoey J, Horton R (2004). Clinical trial registration: a statement from the International Committee of Medical Journal Editors. JAMA.

[41] Korevaar DA, Bossuyt PM, Hooft L (2014). Infrequent and incomplete registration of test accuracy studies: analysis of recent study reports. BMJ Open.

