# Supplementary figures and images for: Small-study effects and time trends in diagnostic test accuracy meta-analyses: a meta-epidemiological study
Source: Syst Rev. 2015 May 9;4:66. doi: 10.1186/s13643-015-0049-8 (PMC4450491; doi:10.1186/s13643-015-0049-8)

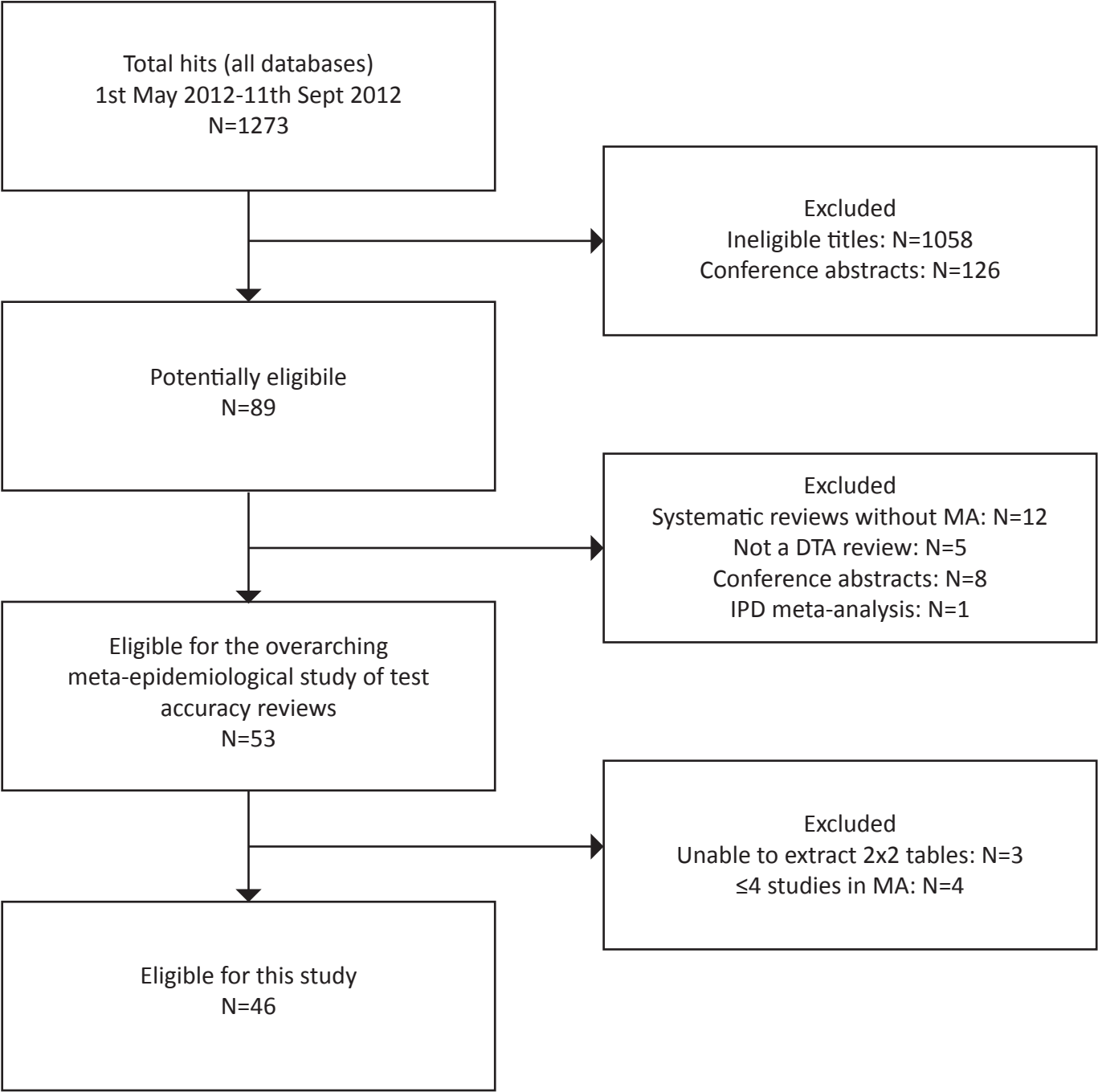

Supplement: Additional file 2: — Inclusion flow chart. Flow chart showing how reviews were included in this study. [file 13643_2015_49_MOESM2_ESM.pdf]
