# Supplementary material for: Small-study effects and time trends in diagnostic test accuracy meta-analyses: a meta-epidemiological study
Source: Syst Rev. 2015 May 9;4:66. doi: 10.1186/s13643-015-0049-8 (PMC4450491; doi:10.1186/s13643-015-0049-8)
Supplement: Additional file 3: — List of included reviews. References of reviews included in this study. [file 13643_2015_49_MOESM3_ESM.docx]

Additional file 3 List of included reviews

1. Al-Sukhni E, Milot L, Fruitman M, Beyene J, Victor JC, Schmocker S, Brown G, McLeod R, Kennedy E. Diagnostic accuracy of MRI for assessment of T category, lymph node metastases, and circumferential resection margin involvement in patients with rectal cancer: a systematic review and meta-analysis. Annals of Surgical Oncology 2012 July;19(7):2212-23.
2. Alldred SK, Deeks JJ, Guo B, Neilson JP, Alfirevic Z. Second trimester serum tests for Down's Syndrome screening. Cochrane Database Syst Rev 2012;6:CD009925.
3. Banerjee A, Newman DR, Van den Bruel A, Heneghan C. Diagnostic accuracy of exercise stress testing for coronary artery disease: a systematic review and meta-analysis of prospective studies. [Review]. International Journal of Clinical Practice 2012 May;66(5):477-92.
4. Beynon R, Sterne JA, Wilcock G, Likeman M, Harbord RM, Astin MP, Burke M, Bessell A, Ben-Shlomo Y, Hawkins J, Hollingworth W, Whiting PF. Is MRI better than CT for detecting a vascular component to dementia? A systematic review and meta-analysis. BMC Neurol 2012 June 6;12(1):33.
5. Chang K, Lu W, Wang J, Zhang K, Jia S, Li F, Deng S, Chen M. Rapid and effective diagnosis of tuberculosis and rifampicin resistance with Xpert MTB/RIF assay: a meta-analysis. J Infect 2012 June;64(6):580-8.
6. Chen J, Yang R, Lu Y, Xia Y, Zhou H. Diagnostic accuracy of endoscopic ultrasound-guided fine-needle aspiration for solid pancreatic lesion: a systematic review. Journal of Cancer Research & Clinical Oncology 2012 September;138(9):1433-41.
7. Chen C, Yang Z, Li Z, Li L. Accuracy of several cervical screening strategies for early detection of cervical cancer: A meta-analysis. International Journal of Gynecological Cancer 2012 July;22(6):908-21.
8. Cheng X, Li Y, Liu B, Xu Z, Bao L, Wang J. 18F-FDG PET/CT and PET for evaluation of pathological response to neoadjuvant chemotherapy in breast cancer: a meta-analysis. Acta Radiologica 2012 July 1;53(6):615-27.
9. De Jong MC, Genders TSS, Van GRJ, Moelker A, Hunink MGM. Diagnostic performance of stress myocardial perfusion imaging for coronary artery disease: A systematic review and meta-analysis. European Radiology 2012 September;22(9):1881-95.
10. Diel R, Loddenkemper R, Nienhaus A. Predictive value of interferon- release assays and tuberculin skin testing for progression from latent TB infection to disease state: A meta-analysis. Chest 2012 July;142(1):63-75.
11. Evangelista L, Cervino AR, Ghiotto C, Al-Nahhas A, Rubello D, Muzzio PC. Tumor marker-guided PET in breast cancer patients-a recipe for a perfect wedding: a systematic literature review and meta-analysis. [Review]. Clinical Nuclear Medicine 2012 May;37(5):467-74.
12. Fan L, Chen Z, Hao XH, Hu ZY, Xiao HP. Interferon-gamma release assays for the diagnosis of extrapulmonary tuberculosis: a systematic review and meta-analysis. FEMS Immunology & Medical Microbiology 2012 August;65(3):456-66.
13. Jaarsma C, Leiner T, Bekkers SC, Crijns HJ, Wildberger JE, Nagel E, Nelemans PJ, Schalla S. Diagnostic performance of noninvasive myocardial perfusion imaging using single-photon emission computed tomography, cardiac magnetic resonance, and positron emission tomography imaging for the detection of obstructive coronary artery disease: a meta-analysis. J Am Coll Cardiol 2012 May 8;59(19):1719-28.
14. Kiewiet JJ, Leeuwenburgh MM, Bipat S, Bossuyt PM, Stoker J, Boermeester MA. A systematic review and meta-analysis of diagnostic performance of imaging in acute cholecystitis. Radiology 2012 September;264(3):708-20.
15. Kim HP, Vance RB, Shaheen NJ, Dellon ES. The Prevalence and Diagnostic Utility of Endoscopic Features of Eosinophilic Esophagitis: A Meta-analysis. Clinical Gastroenterology & Hepatology 2012 September;10(9):988-96.
16. Kocken M, Uijterwaal MH, de Vries AL, Berkhof J, Ket JC, Helmerhorst TJ, Meijer CJ. High-risk human papillomavirus testing versus cytology in predicting post-treatment disease in women treated for high-grade cervical disease: a systematic review and meta-analysis. [Review]. Gynecologic Oncology 2012 May;125(2):500-7.
17. Li C, Su N, Yang X, Yang X, Shi Z, Li L. Ultrasonography for detection of disc displacement of temporomandibular joint: a systematic review and meta-analysis. [Review]. Journal of Oral & Maxillofacial Surgery 2012 June;70(6):1300-9.
18. Lin CY, Chen JH, Liang JA, Lin CC, Jeng LB, Kao CH. 18F-FDG PET or PET/CT for detecting extrahepatic metastases or recurrent hepatocellular carcinoma: A systematic review and meta-analysis. European Journal of Radiology 2012 September;81(9):2417-22.
19. Lu Y-Y, Chen J-H, Liang J-A, Wang H-Y, Lin C-C, Lin W-Y, Kao C-H. Clinical value of FDG PET or PET/CT in urinary bladder cancer: A systemic review and meta-analysis. European Journal of Radiology 2012 September;81(9):2411-6.
20. Mavromatis ID, Antonopoulos CN, Matsoukis IL, Frangos CC, Skalkidou A, Creatsas G, Petridou ET. Validity of intraoperative gross examination of myometrial invasion in patients with endometrial cancer: a meta-analysis. Acta Obstetricia et Gynecologica Scandinavica 2012 July;91(7):779-93.
21. Morris RK, Riley RD, Doug M, Deeks JJ, Kilby MD. Diagnostic accuracy of spot urinary protein and albumin to creatinine ratios for detection of significant proteinuria or adverse pregnancy outcome in patients with suspected pre-eclampsia: systematic review and meta-analysis. BMJ 2012;345:e4342.
22. Neto AS, Nassar AP, Jr., Cardoso SO, Manetta JA, Pereira VG, Esposito DC, Damasceno MC, Slooter AJ. Delirium screening in critically ill patients: a systematic review and meta-analysis. [Review]. Critical Care Medicine 2012 June;40(6):1946-51.
23. Pai NP, Balram B, Shivkumar S, Martinez-Cajas JL, Claessens C, Lambert G, Peeling RW, Joseph L. Head-to-head comparison of accuracy of a rapid point-of-care HIV test with oral versus whole-blood specimens: A systematic review and meta-analysis. The Lancet Infectious Diseases 2012 May;12(5):373-80.
24. Qu X, Huang X, Yan W, Wu L, Dai K. A meta-analysis of (1)(8)FDG-PET-CT, (1)(8)FDG-PET, MRI and bone scintigraphy for diagnosis of bone metastases in patients with lung cancer. Eur J Radiol 2012 May;81(5):1007-15.
25. Romero J, Xue X, Gonzalez W, Garcia MJ. CMR imaging assessing viability in patients with chronic ventricular dysfunction due to coronary artery disease: a meta-analysis of prospective trials. Jacc: Cardiovascular Imaging 2012 May;5(5):494-508.
26. Sadeghi R, Gholami H, Zakavi SR, Kakhki VR, Horenblas S. Accuracy of 18F-FDG PET/CT for diagnosing inguinal lymph node involvement in penile squamous cell carcinoma: systematic review and meta-analysis of the literature. Clin Nucl Med 2012 May;37(5):436-41.
27. Sadigh G, Carlos RC, Neal CH, Dwamena BA. Ultrasonographic differentiation of malignant from benign breast lesions: A meta-analytic comparison of elasticity and BIRADS scoring. Breast Cancer Research and Treatment 2012 May;133(1):23-35.
28. Sandroni C, Cavallaro F, Marano C, Falcone C, De SP, Antonelli M. Accuracy of plethysmographic indices as predictors of fluid responsiveness in mechanically ventilated adults: a systematic review and meta-analysis. Intensive Care Medicine 2012 September;38(9):1429-37.
29. Shang Y, Ju W, Kong Y, Schroder PM, Liang W, Ling X, Guo Z, He X. Performance of polymerase chain reaction techniques detecting perforin in the diagnosis of acute renal rejection: a meta-analysis. PLoS ONE [Electronic Resource] 2012;7(6):e39610.
30. Shen Y-C, Liu M-Q, Wan C, Chen L, Wang T, Wen F-Q. Diagnostic accuracy of vascular endothelial growth factor for malignant pleural effusion: A meta-analysis. Experimental and Therapeutic Medicine 2012 June;3(6):1072-6.
31. Singh B, Parsaik AK, Agarwal D, Surana A, Mascarenhas SS, Chandra S. Diagnostic accuracy of pulmonary embolism rule-out criteria: a systematic review and meta-analysis. [Review]. Annals of Emergency Medicine 2012 June;59(6):517-20.
32. Smith TO, Lewis M, Song F, Toms AP, Donell ST, Hing CB. The diagnostic accuracy of anterior cruciate ligament rupture using magnetic resonance imaging: A meta-analysis. European Journal of Orthopaedic Surgery and Traumatology 2012 May;22(4):315-26.
33. Smith TO, Drew B, Toms AP, Jerosch-Herold C, Chojnowski AJ. Diagnostic accuracy of magnetic resonance imaging and magnetic resonance arthrography for triangular fibrocartilaginous complex injury: a systematic review and meta-analysis. [Review]. Journal of Bone & Joint Surgery - American Volume 2012 May 2;94(9):824-32.
34. Smith TO, Drew BT, Toms AP. A meta-analysis of the diagnostic test accuracy of MRA and MRI for the detection of glenoid labral injury. Archives of Orthopaedic and Trauma Surgery 2012 July;132(7):905-19.
35. Tai T-W, Wu C-Y, Su F-C, Chern T-C, Jou I-M. Ultrasonography for Diagnosing Carpal Tunnel Syndrome: A Meta-Analysis of Diagnostic Test Accuracy. Ultrasound in Medicine and Biology 2012 July;38(7):1121-8.
36. Tashakkor AY, Nicolaou S, Leipsic J, Mancini GB. The Emerging Role of Cardiac Computed Tomography for the Assessment of Coronary Perfusion: A Systematic Review and Meta-analysis. Canadian Journal of Cardiology 2012 July;28(4):413-22.
37. Thangaratinam S, Brown K, Zamora J, Khan KS, Ewer AK. Pulse oximetry screening for critical congenital heart defects in asymptomatic newborn babies: a systematic review and meta-analysis. Lancet 2012 May 1.
38. Treglia G, Castaldi P, Rindi G, Giordano A, Rufini V. Diagnostic performance of Gallium-68 somatostatin receptor PET and PET/CT in patients with thoracic and gastroenteropancreatic neuroendocrine tumours: A meta-analysis. Endocrine 2012 August;42(1):80-7.
39. Underwood M, Arbyn M, Redman C, Smith WP. Accuracy of colposcopic directed punch biopsies: A systematic review and meta-analysis. BJOG: An International Journal of Obstetrics and Gynaecology 2012 June;Conference(var.pagings):163.
40. van Teeffelen AS, Van Der Heijden J, Oei SG, Porath MM, Willekes C, Opmeer B, Mol BW. Accuracy of imaging parameters in the prediction of lethal pulmonary hypoplasia secondary to mid-trimester prelabor rupture of fetal membranes: a systematic review and meta-analysis. Ultrasound in Obstetrics & Gynecology 2012 May;39(5):495-9.
41. Wang Z, Dong ZY, Chen JQ, Liu JL. Diagnostic value of sentinel lymph node biopsy in gastric cancer: a meta-analysis. [Review]. Annals of Surgical Oncology 2012 May;19(5):1541-50.
42. Webb RC, Howard RS, Stojadinovic A, Gaitonde DY, Wallace MK, Ahmed J, Burch HB. The utility of serum thyroglobulin measurement at the time of remnant ablation for predicting disease-free status in patients with differentiated thyroid cancer: a meta-analysis involving 3947 patients. Journal of Clinical Endocrinology & Metabolism 2012 August;97(8):2754-63.
43. Wu L, Dai ZY, Qian YH, Shi Y, Liu FJ, Yang C. Diagnostic Value of Serum Human Epididymis Protein 4 (HE4) in Ovarian Carcinoma: A Systematic Review and Meta-Analysis. International Journal of Gynecological Cancer 2012 September;22(7):1106-12.
44. Wu L-M, Gu H-Y, Qu X-H, Zheng J, Zhang W, Yin Y, Xu J-R. The accuracy of ultrasonography in the preoperative diagnosis of cervical lymph node metastasis in patients with papillary thyroid carcinoma: A meta-analysis. European Journal of Radiology 2012 August;81(8):1798-805.
45. Wu L-M, Hu J-N, Hua J, Liu M-J, Chen J, Xu J-R. Diagnostic value of diffusion-weighted magnetic resonance imaging compared with fluorodeoxyglucose positron emission tomography/computed tomography for pancreatic malignancy: A meta-analysis using a hierarchical regression model. Journal of Gastroenterology and Hepatology 2012 June;27(6):1027-35.
46. Zhao L, He Z-Y, Zhong X-N, Cui M-L. 18FDG-PET/CT for detection of mediastinal nodal metastasis in non-small cell lung cancer: A meta-analysis. Surgical Oncology 2012 September;21(3):230-6.
